# Vitamin D alleviates intracerebral hemorrhage symptoms by modulating pro-inflammatory/anti-inflammatory phenotypic transformation of microglia through P2X7R activation of NLRP3

**DOI:** 10.1016/j.ibneur.2026.06.015

**Published:** 2026-06-23

**Authors:** Xiujun Zhang, Bensi Zhang, Chun Shi, Natnicha Thammarangsee, Waleephan Treebupachatsakul, Rungusa Pantan, Suteera Narakornsak, Manussabhorn Phatsara

**Affiliations:** a Department of Anatomy, Faculty of Medicine, Chiang Mai University, Inthawarorot Road, Chiang Mai, Thailand; b Department of Human Anatomy, College of Basic Medicine, Xiaguan Campus, Dali University, Wanhua Road, Dali, Yunnan, China; c College of Dental Medicine, Western University of Health Sciences, Pomona, CA, USA

**Keywords:** Vitamin D, Intracerebral hemorrhage, P2X7R, NLRP3, Pro-inflammatory/anti-inflammatory phenotypic transformation

## Abstract

**Objective:**

The primary objective of this study was to analyze the potential influence of vitamin D on microglial phenotypic transformation in cerebral hemorrhage.

**Methods:**

To simulate cerebral hemorrhage conditions, a hemin-induced brain hemorrhage cell model was established, and cell viability was subsequently measured by CCK-8 assay. Western blotting was then performed to detect the expression of P2X7R, NLRP3, and microglial phenotypic transformation marker proteins. The expression of CD16/32 and CD206 was assessed by immunofluorescence, and ELISA was used to measure levels of inflammatory cytokines, including IL-4, TNF-α, IL-1β, IL-10, and IL-6.

**Results:**

Vitamin D exhibited a stimulating effect on cell proliferation, reduced the expression of CD16/32 and iNOS, and boosted the expression of CD206, Arg-1, and Iba-1. Moreover, vitamin D inhibited TNF-α, IL-1β, and IL-10 levels and elevated IL-4 and IL-6 levels. This implies that vitamin D can suppress pro-inflammatory phenotypic transformation and promote anti-inflammatory phenotypic transformation in microglia. Additionally, vitamin D suppressed the expression of P2X7R and NLRP3. The effect of P2X7R inhibitor (A438079) treatment was comparable to that of vitamin D, with the addition of vitamin D further enhancing the effect.

**Conclusion:**

Vitamin D was found to regulate microglial phenotypic transformation through the P2X7R/NLRP3 signaling pathway, thereby promoting anti-inflammatory and inhibiting pro-inflammatory phenotypic transformation. This finding may lead to new therapeutic strategies for the treatment of ICH-related brain injury.

## 1 Introduction

Globally, intracerebral hemorrhage (ICH) accounts for roughly 10–15% of all stroke cases and represents a significant neurological emergency that poses a major threat to survival. Among those affected, the first-month mortality rate can reach as high as 40%, with survivors often left with long-lasting disabilities (Siddiqui et al., 2017, Yogendrakumar et al., 2018). To date, there are no established pharmacological or surgical treatments that can effectively improve the prognosis of ICH (Mendelow et al., 2013). Microglia are vital immune cells in the brain and play a crucial role in various acute brain injuries (Koichiro et al., 2024). During a brain injury, the classical and alternative activation states of individual microglia can influence tissue damage and its subsequent repair process (Nan et al., 2022). Inflammation is regulated by both pro-inflammatory and anti-inflammatory microglia (Guo et al., 2022). Following an ICH, modulation of microglial function is expected to attenuate ICH-associated brain damage, thereby promoting tissue repair and functional recovery (Zhang et al., 2017). Therefore, the search for drugs that can regulate microglia function is urgent.

Vitamin D is a neuroprotective hormone that regulates diverse genomic and non-genomic pathways (Jamali et al., 2018, Hii and Ferrante, 2016). In connection with brain disorders, vitamin D has been shown to be beneficial for animals afflicted with ischemic stroke and subarachnoid hemorrhage by maintaining the integrity of the blood–brain barrier (Guo et al., 2018, Enkhjargal et al., 2017). Additionally, vitamin D has been found to enhance hematoma clearance and facilitate neurological recovery after an ICH (Liu et al., 2022a). Moreover, it has been discovered that vitamin D can suppress pro-inflammatory microglia and enhance the growth of anti-inflammatory microglia to alleviate neuroinflammation in Parkinson’s disease (Calvello et al., 2017). However, it remains unclear whether vitamin D affects the phenotypic transformation of microglia in ICH.

P2X7R (Purinergic Receptor P2X7) is an ionotropic purinergic receptor. In the central nervous system, P2X7R is mainly expressed in microglia (Skaper et al., 2010). Several studies have shown that P2X7R plays a key role in regulating microglial phenotypic transformation (Takenouchi et al., 2009, Young et al., 2015). The NLR family, which contains three members with a pyrin structural domain, includes NLRP3 (NOD-like receptor family pyrin domain-containing protein 3), the most extensively studied member of the NOD-like receptor family. NLRP3 is a downstream target of P2X7R, and increased expression of P2X7R can activate NLRP3 (Di Virgilio et al., 2017). For example, the increased expression of P2X7R/NLRP3 in Alzheimer’s disease promotes microglia to undergo pro-inflammatory phenotypic transformation and aggravates neuroinflammation (Zhang et al., 2022a). P2X7R/NLRP3 is also involved in the phenotypic transformation of microglia to a pro-inflammatory type in hippocampal neuron injury (Liu et al., 2022a). At present, most of the studies on P2X7R/NLRP3 focus on spinal cord injury and neuropathic pain (Fan et al., 2020, Hu et al., 2020). Whether P2X7R/NLRP3 affects the phenotypic transformation of microglia in ICH is unclear.

In the present study, we employed hemin as a microglial-inducing agent to establish a hemorrhagic model to assess the impact of hemin on the expression levels of P2X7R and NLRP3, as well as the phenotypic transformation profile of microglia. We subsequently introduced vitamin D and P2X7R antagonists to assess their effects on hemin-mediated microglial phenotypic transformation. Additionally, we assessed whether vitamin D exerts a regulatory influence on the expression of P2X7R and NLRP3. The findings from our study suggest that vitamin D can suppress the P2X7R/NLRP3 signaling pathway, thereby inhibiting the shift of microglia toward a pro-inflammatory phenotype.

## 2 Materials and methods

### 2.1 Reagents

The mouse BV2 microglial cell line was obtained from the Chinese Academy of Medical Sciences (Beijing, China). Modified DMEM (Dulbecco’s Modified Eagle Medium‌) (Cat# SH30243.01) and fetal bovine serum (FBS) (Cat# SH30070.03) were purchased from HyClone Inc. (USA). Hemin (Cat# 51280–25 G), TRIzol reagent (Cat# 15596026), and penicillin-streptomycin (Cat# 15140122) were sourced from Sigma-Aldrich (USA) or Invitrogen (USA). Vitamin D (1,25-dihydroxyvitamin D3) was purchased from Merck (Darmstadt, Germany, Cat# 32222–06–3). It was utilized for cell treatment. The P2X7R inhibitor A438079 (Cat# HY−15488) was obtained from MedChemExpress (MCE, China). Paraformaldehyde (4%) and Triton X−100 (0.1%) were purchased from Abcam (USA). For cell viability and protein analysis, the CCK−8 was purchased from Boster (China, Cat# AR1199) and Beyotime (China, Cat# C0048/C1052). RIPA lysis buffer was from Pierce (USA, Cat# 89900) or Beyotime (China, Cat# P0013B), and the BCA protein assay kit was from Thermo Fisher Scientific (MA, USA, Cat# 23225/23250/A55864). Primary antibodies against iNOS (Cat# sc−7271), Arg−1 (Cat# 93668S), Iba−1 (Cat# ab283319), P2X7R (Cat# ab109054/ab195356), NLRP3 (Cat# ab214185), and GAPDH (Cat# 5174S), as well as immunofluorescence antibodies (anti-CD206, anti-CD16/32, and anti-Iba−1), were purchased from Abcam (USA), Santa Cruz (USA), or Cell Signaling Technology (USA). 4’,6-diamidino−2-phenylindole (DAPI) staining solution was obtained from Sigma-Aldrich (Cat# D1306). The ECL chemiluminescence kit was sourced from Thermo Scientific (MA, USA, Cat# 32106). For molecular biology assays, the ServiceBio® RT First Strand cDNA Synthesis Kit and specific primers were provided by ServiceBio (Wuhan, China). ELISA kits for quantifying tumor necrosis factor-α (TNF-α), interleukin−1α (IL−1α), IL−6, IL−4, and IL−10 were purchased from Elabscience Biotechnology (Wuhan, China).

### 2.2 Cell culture

BV2 microglia were sourced from the Chinese Academy of Medical Sciences (Beijing, China). The cells were then grown in modified DMEM (HyClone Inc., USA) enriched with 10% FBS, 100 U/mL penicillin, and 100 U/mL streptomycin. Next, BV2 cells were treated with hemin (Sigma-Aldrich, USA) solutions of 0 μM, 25 μM, 50 μM, 75 μM, 100 μM, and 125 μM to simulate an in vitro model of ICH. Once stimulated, cell viability was measured using a cell counting kit (CCK−8, Cell Counting Kit−8‌, Boster, China) to identify the optimal heme concentration for use in the ICH model. A portion of the cultured cells was used as the NC (normal cultured mouse BV2 microglial cells without undergoing cell modeling experiments) group and served as the control group for the experiment.

### 2.3 Cell treatment

Randomly dividing the cells into four groups, as follows, the NC group received no hemin treatment; the Hemin group received 100 μM hemin; the Vitamin D group received pre-treatment with 100 μM vitamin D for 12 h, followed by hemin induction; the P2X7R inhibitor (A438079) group received pre-treatment with 10 μM A438079 (MCE, China) for 30 min, followed by hemin induction; the Vitamin D + P2X7R inhibitor group received pre-treatment with 10 μM A438079 for 30 min, followed by addition of 100 μM vitamin D for 12 h, and then hemin induction.

### 2.4 Cell viability

Cell proliferation was assessed using the CCK−8 kit (Beyotime, China). The cells (1 ×10^4^ cells/well) were seeded in a 96-well plate and subjected to various treatments depending on the experimental group. Following the treatment, 10 μL/well of the CCK−8 solution was added to each well. The plate was then incubated at 37 °C for 3 h. Absorbance was subsequently measured at a wavelength of 450 nm using an enzyme marker.

### 2.5 Enzyme-linked immunosorbent assay (ELISA)

TNF-α, IL−1β, IL−6, IL−4, and IL−10 levels were quantified using an enzyme-linked immunosorbent assay (ELISA) kit from Elabscience Biotechnology (Wuhan, China).

### 2.6 Western blot

The proteins were isolated by centrifugation after lysing the cells with a RIPA (Radio Immunoprecipitation Assay) lysis buffer (Pierce, USA). The protein concentration was determined using a BCA (Bicinchoninic Acid) protein assay kit (Thermo Fisher Scientific, MA, USA). Equal amounts of protein were then loaded onto a 12% SDS-PAGE gel and electrophoresed before being transferred to a PVDF (Polyvinylidene Difluoride) membrane. The membrane was then sealed with 5% skimmed milk and incubated with primary antibodies at 4 °C overnight. The primary antibodies included iNOS (Inducible nitric oxide synthase), Arg−1 (Arginase−1), Iba−1 (Ionized calcium-binding adapter molecule 1), P2X7R, NLRP3, and GAPDH (Glyceraldehyde−3-phosphate dehydrogenase‌). Finally, the membranes were incubated with the corresponding secondary antibodies for 1 h at room temperature. The ECL (Enhanced chemiluminescence) kit (Thermo Scientific, Massachusetts, USA) was then used for analysis.

### 2.7 Immunofluorescence

The cells were divided into different groups and treated accordingly. Subsequently, the cells were placed on a glass slide, fixed with 4% paraformaldehyde, and treated with 0.1% Triton X−100 (Abcam, USA) for 20 min to permeabilize the cells. Afterward, the cells were probed with anti-mouse CD206 (Abcam, USA) or anti-mouse CD16/32 (Abcam, USA) antibodies at dilutions of 1:200 and 1:100, respectively. After 24 h of incubation, the cells were washed with PBS and then treated with anti-mouse Iba−1 (Abcam, USA) antibodies at a dilution of 1:100 at 4 °C overnight. The cells were washed again with PBS and then incubated with secondary antibody for 3 h at room temperature. Finally, cells were stained with DAPI for 5 min at 37 °C and imaged under a confocal microscope (Olympus, Tokyo, Japan).

### 2.8 RT-qPCR

Total RNA was extracted from cells using TRIzol reagent (Sigma-Aldrich, USA) and then reverse-transcribed using the ServiceBio® RT First Strand cDNA Synthesis Kit (ServiceBio, Wuhan, China) according to the manufacturer’s instructions. Specific primer sequences are presented in Table 1. GAPDH was utilized as an internal reference to quantify messenger RNA (mRNA) levels.

**Table 1:** Primer sequence information.

**Gene name**	**Forward primer (5′–3′)**	**Reverse primer (5′–3′)**
**P2X7R**	5′-GAACAATATCGACTTCCCCGG−3′	5′-TTATCGCCTGTTTCTCGGAAG−3′
**NLRP3**	5′-ATTACCCGCCCGAGAAAGG−3′	5′-ATTACCCGCCCGAGAAAGG−3′
**GAPDH**	5′-CTTCACCACCATGGAGAAGGC−3′	5′-GGCATGGACTGTGGTCATGAG−3′

### 2.9 Statistical analysis

The mean ± standard deviation of three independent experiments in triplicate are provided. Statistical analyses were performed using GraphPad Prism 7 software, in which an independent-samples *t*-test was used to compare data between two groups, and a one-way analysis of variance (ANOVA) was used to evaluate differences among multiple groups. A *p*-value < 0.05 indicates that the difference is statistically significant.

## 3 Results

### 3.1 Hemin induces microglial phenotypic transformation

**Fig. 1:**
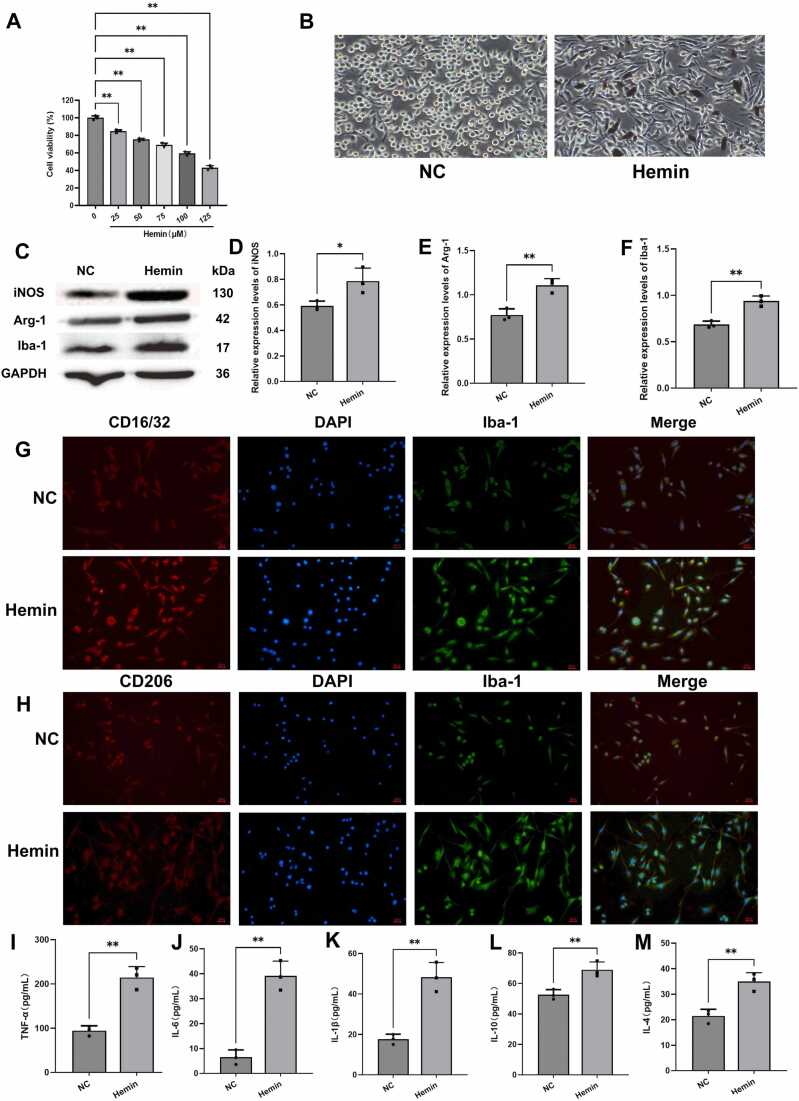
Hemin induces microglial phenotypic transformation toward pro-inflammatory/anti-inflammatory type. A: CCK−8 to detect cell viability; B: Microscopic observation of cell morphology; C, D, E, and F: Western blotting to detect iNOS, Arg−1 and Iba−1 proteins; Immunofluorescence detection of CD16/32 (G) and CD206 expression (H); I, J, K, L, and M: ELISA to detect the concentrations of TNF-α, IL−6, IL−1β, IL−10, and IL−4, respectively. In summary, hemin can drive microglial phenotypic transformation toward pro-inflammatory/anti-inflammatory; cells were stained with DAPI. The number of independent biological replicates for each experiment is 3. Scale bar: 20 μm. ** *p* < 0.01, * *p* < 0.05.

Based on cell viability measurements after treatment with various hemin concentrations, cell viability decreased in a concentration-dependent manner. However, at a concentration of 100 μM, the viability fell to a relatively low level of 59% (Fig. 1A). Consequently, we opted to utilize 100 μM for subsequent experimental evaluations. Through microscopic observation of cell morphology, we discovered that resting microglia were small and round. However, upon hemin treatment, the microglia exhibited amoeba-like characteristics (Fig. 1B). Western blotting analysis indicated an increase in the expression of iNOS, Arg−1, and Iba−1 post-hemin induction (Fig. 1C-F). Furthermore, immunofluorescence studies confirmed that hemin induction stimulated the expression of CD16/32 and CD206 (Fig. 1G, H). We assessed the expression of pro-inflammatory factors (TNF-α, IL−6, and IL−1β) and anti-inflammatory factors (IL−10 and IL−4) by ELISA. We found that after hemin treatment, both the expression of pro-inflammatory and anti-inflammatory factors were upregulated, with a greater increase in pro-inflammatory factors (Fig. 1I-M). In summary, hemin can drive microglial phenotypic transformation toward pro-inflammatory/anti-inflammatory states.

### 3.2 Hemin upregulated the expression of P2X7R/NLRP3 in microglia, thereby regulating the P2X7R/NLRP3 pathway

**Fig. 2:**
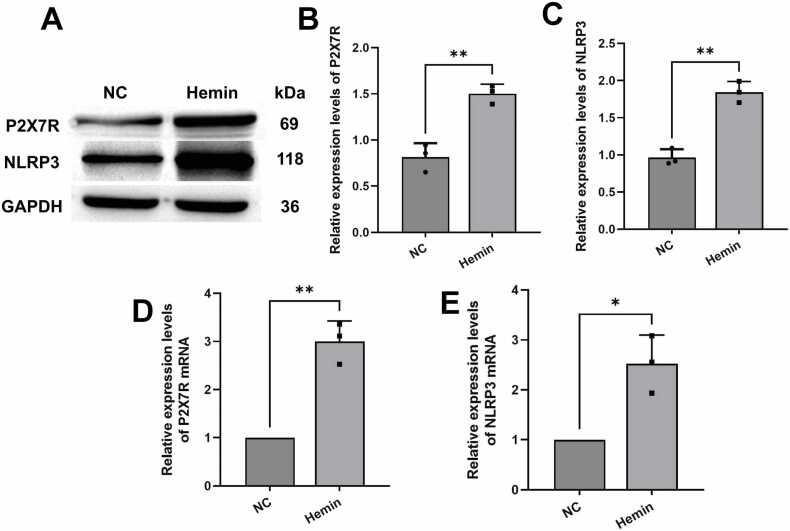
Hemin regulates the microglial P2X7R/ /NLRP3 pathway. A, B, and C: Western blot detects protein expression of P2X7R and NLRP3; D and E: RT-qPCR detects mRNA expression of P2X7R and NLRP3. In summary, these findings suggest that hemin regulates the P2X7R/NLRP3 pathway. The number of independent biological replicates for each experiment is 3. ** *p* < 0.01, *p < 0.05.

The purpose of this study was to determine whether hemin affects the P2X7R/NLRP3 pathway. We utilized western blotting and RT-qPCR to assess the protein and mRNA expression of P2X7R and NLRP3, respectively. Our results revealed that hemin significantly enhanced both the protein (Fig. 2A-C) and mRNA (Fig. 2D, E) expression of P2X7R and NLRP3. These findings suggest that hemin upregulated the P2X7R/NLRP3 expression and regulated the P2X7R/NLRP3 pathway.

### 3.3 Vitamin D can regulate the phenotypic transformation of microglia to a pro-inflammatory/anti-inflammatory type and protect microglia from the influence of hemin

**Fig. 3:**
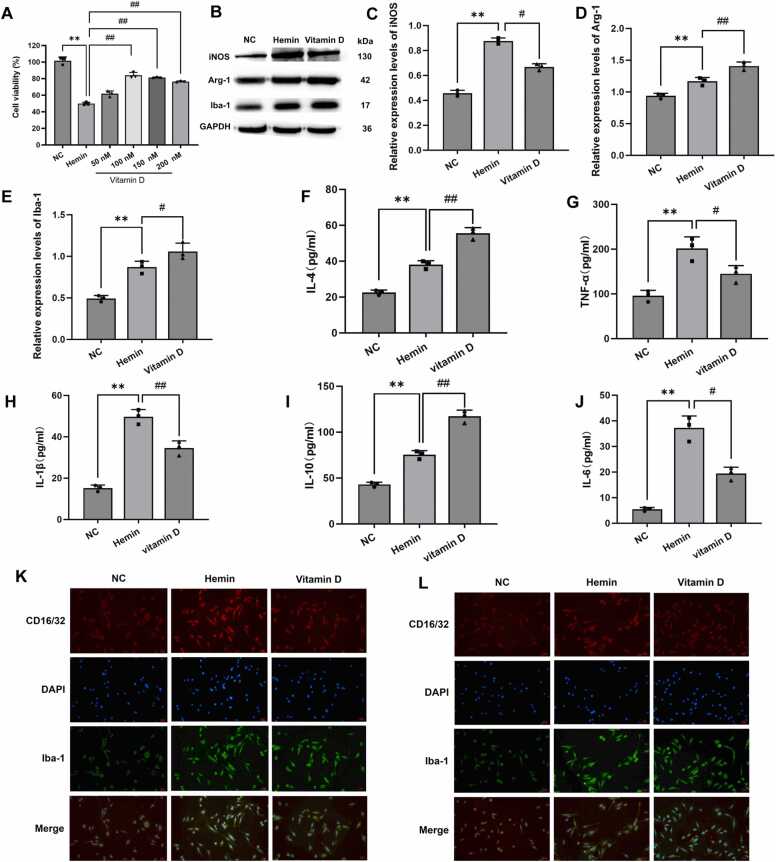
Vitamin D can regulate the phenotypic transformation of microglia to pro-inflammatory/anti-inflammatory type and protect microglia from the influence of hemin. A: CCK−8 to detect cell viability; B, C, D, and E: Western blot was used to detect the expression of iNOS, Arg−1, and Iba−1; F, G, H, I, and J: ELISA to detect the concentrations of IL−4, TNF-α, IL−1β, IL−10, and IL−6, respectively; Immunofluorescence detection of CD16/32 (K) and CD206 (L) expression. In summary, these findings suggest that vitamin D promotes microglial phenotypic transformation toward the anti-inflammatory type and inhibits their phenotypic transformation toward the pro-inflammatory type; cells were stained with DAPI. The number of independent biological replicates for each experiment is 3. Scale bar: 20 μm. ## *p* < 0.01, ** *p* < 0.01, # *p* < 0.05.

In this study, we aimed to investigate the influence of vitamin D on microglial phenotypic transformation. Using the CCK−8 assay, we observed that microglial viability was significantly improved across various concentrations of vitamin D, with 100 μM vitamin D yielding the most favorable effect (Fig. 3A). For further exploration, we selected 100 μM vitamin D for subsequent experiments. Additionally, we examined the protein expression of iNOS, Arg−1, and Iba−1. Protein blotting revealed a significant decline in iNOS expression post-vitamin D treatment, whereas the expression of Arg−1 and Iba−1 increased significantly in comparison to the Hemin group (Fig. 3B-E). Concentrations of pro-inflammatory cytokines TNF-α, IL−6, and IL−1β significantly decreased, while the concentration of anti-inflammatory cytokines IL−10 and IL−4 significantly increased, when compared to the Hemin group (Fig. 3F-J). Lastly, immunofluorescence results showed a significant decrease in CD16/32 expression after vitamin D treatment (Fig. 3K), whereas CD206 expression increased significantly (Fig. 3L) compared with the Hemin group. These findings suggest that vitamin D promotes microglial phenotypic transformation toward the anti-inflammatory type and inhibits their phenotypic transformation toward the pro-inflammatory type.

### 3.4 Vitamin D inhibits the P2X7R/ /NLRP3 pathway

**Fig. 4:**
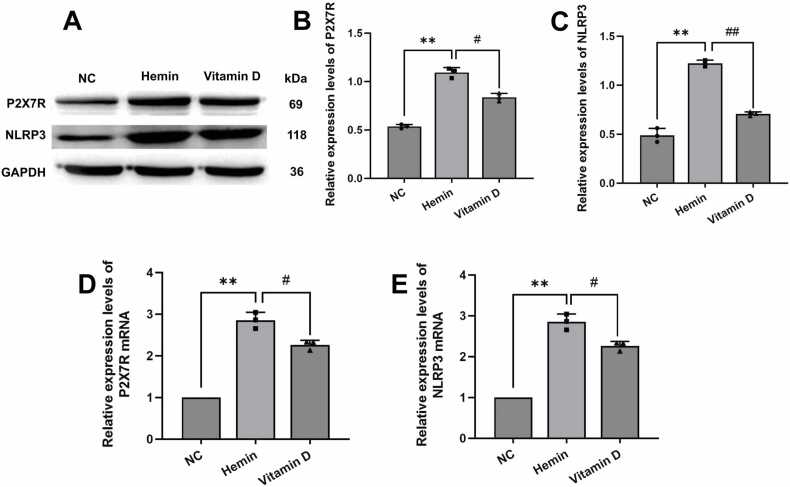
Vitamin D inhibits the P2X7R/ /NLRP3 pathway. A, B, and C: Western blot detects protein expression of P2X7R and NLRP3; D and E: RT-qPCR detects mRNA expression of P2X7R and NLRP3. The number of independent biological replicates for each experiment is 3. Thus, it is clear that vitamin D can inhibit the P2X7R/NLRP3 signaling pathway. ## *p* < 0.01, ** *p* < 0.01, # *p* < 0.05.

To assess the regulatory effect of vitamin D on the P2X7R/NLRP3 signaling pathway, we utilized western blot and RT-qPCR analyses. These analyses revealed that vitamin D significantly repressed the protein and mRNA expression of P2X7R and NLRP3, respectively, compared with hemin (Fig. 4A-E). Thus, it is clear that vitamin D can inhibit the P2X7R/NLRP3 signaling pathway.

### 3.5 Vitamin D modulates the pro-inflammatory/anti-inflammatory-type phenotypic transformation of microglia through the P2X7R/NLRP3 pathway

**Fig. 5:**
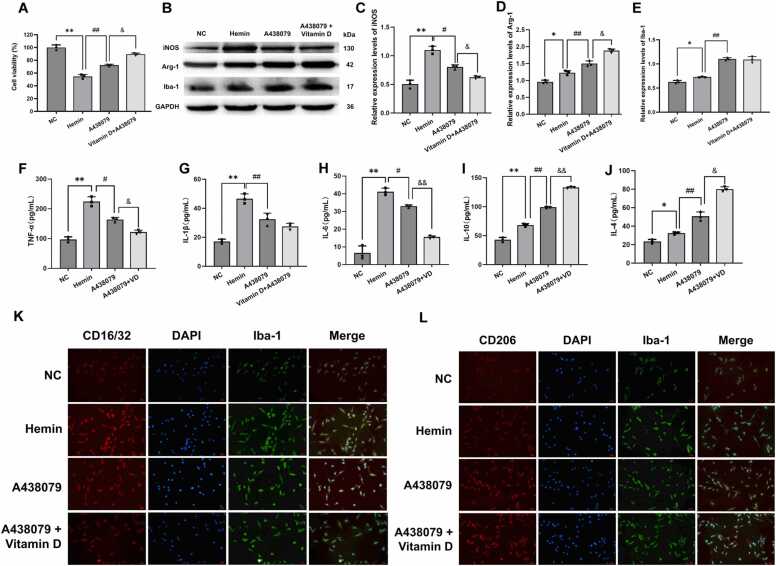
Vitamin D modulates the pro-inflammatory/anti-inflammatory-type phenotypic transformation of microglia through the P2X7R/NLRP3 pathway. A: CCK−8 to detect cell viability; B, C, D, and E: Western blot was used to detect the expression of iNOS, Arg−1, and Iba−1; F, G, H, I, and J: ELISA to detect the concentrations of TNF-α, IL−1β, IL−6, IL−10, and IL−4, respectively; Immunofluorescence detection of CD16/32 (K) and CD206 expression (L). To summarize, vitamin D can promote the activation of anti-inflammatory microglia and simultaneously inhibit the development of pro-inflammatory microglia by suppressing the P2X7R/NLRP3 signaling pathway; cells were stained with DAPI. The number of independent biological replicates for each experiment is 3. Scale bar: 20μm. ## *p* < 0.01, ** *p* < 0.01, # *p* < 0.05.

We next investigated whether vitamin D regulates pro-inflammatory/anti-inflammatory phenotypic transformation in microglia through the P2X7R/NLRP3 signaling pathway. The CCK−8 viability assay showed a significant increase in cell viability with A438079 treatment compared with the Hemin group, a trend further amplified by vitamin D (Fig. 5A). Furthermore, the protein blotting assay showed that A438079 notably suppressed iNOS expression while enhancing Arg−1 and Iba−1 expression, with vitamin D further augmenting the effect of A438079 (Fig. 5B-E). A438079 treatment notably reduced the levels of TNF-α, IL−6, and IL−1β while elevating the concentration of IL−10 and IL−4, and vitamin D further enhanced the impact of A438079 (Fig. 5F-J). Simultaneously, similar results were obtained with immunofluorescence detection of CD16/32 (Fig. 5K) and CD206 (Fig. 5L) expression. To summarize, vitamin D can promote the activation of anti-inflammatory microglia and simultaneously inhibit the development of pro-inflammatory microglia by suppressing the P2X7R/NLRP3 signaling pathway.

## 4 Discussion

Microglial phenotypes and states are diverse and highly heterogeneous, depending on specific environmental conditions (Paolicelli et al., 2022). The inflammatory milieu following intracerebral hemorrhage (ICH) is uniquely complex (Zhang et al., 2022b), featuring erythrocyte-derived products such as hemoglobin, hemin, and iron, along with plasma proteins and thrombin (Sayeed a et al., 2017). Among these, hemin has been shown to activate microglia through multiple parallel pathways, including TLR4/NF-κB signaling (driving pro-inflammatory cytokine production) and PPARγ-dependent transcriptional responses (associated with phagocytic receptor upregulation). This study, therefore, characterized BV2 microglial responses to hemin exposure using a multi-parametric approach based on (1) expression profiles of activation-associated surface markers (CD16/32 and CD206), (2) cytokine/chemokine secretion spectra (IL−1β, TNF-α, IL−6, IL−4, IL−10), and (3) functional correlates. Our findings revealed that hemin-exposed BV2 microglia simultaneously upregulated CD16/32 (a marker associated with Fc receptor-mediated phagocytosis and immune complex recognition) and CD206 (a mannose receptor linked to scavenging and tissue remodeling), along with elevated production of both pro-inflammatory (IL−1β, TNF-α, IL−6) and anti-inflammatory (IL−4, IL−10) cytokines. This mixed secretory and surface phenotype—characterized by concurrent inflammatory and repair-associated features—is consistent with the emerging view that microglial activation in response to hemorrhagic stimuli does not conform to a binary M1/M2 framework but rather follows stimulus-specific transcriptional programs that may include hybrid functional states. We consider the pro-inflammatory/anti-inflammatory phenotypic transformation of microglia to be equivalent to the amoeboid and ramified states.

The proteomic analysis of protein profiles in primary microglial cells and BV2 cells under stimulated conditions (Luan et al., 2022) revealed that, when BV2 cells were exposed to inflammatory stimuli such as lipopolysaccharide (LPS), they could be activated, but the immune response was weakened or altered, with a narrower scope. They even showed higher inflammatory levels, even without stimulation. In our experiments, we also found that BV2 cells are prone to excessive trypsin digestion, so careful operation is required when culturing them; excessive trypsin treatment can easily cause cell damage and change their phenotypic characteristics. The study of BV2 cells derived from human induced pluripotent stem cells revealed that their immune function is affected during lipid degradation (Stöberl et al., 2023). RNA sequencing and functional immune response assays assessed the states of wild-type and mutant BV2 cell lines under basal conditions and following pro-inflammatory LPS activation. Most genes encoding pro-inflammatory cytokines, as well as those involved in phagocytosis, antigen presentation, and T lymphocyte co-stimulation, were differentially overexpressed. The changes in the transcriptome were reflected in changes in phagocytic ability, inflammasome activation, increased release of inflammatory cytokines (including TNF), and upregulation of the response of T lymphocytes activated by peptides presented by mutant BV2 cells. We also recognize that there are certain limitations compared to primary microglia or microglia derived from human induced pluripotent stem cells. We will improve upon these in our subsequent studies.

Scientists have reported that hemin indirectly regulates the expression and function of the P2X7 receptor (P2X7R), primarily through transcriptional control and downstream inflammatory cascades (Jimenez-Mateos et al., 2019). In conditions such as hemorrhagic stroke or sickle cell disease, high levels of hemin trigger the generation of reactive oxygen species (ROS) and cellular stress. This hemin-induced stress strongly upregulates P2X7R expression, which, in turn, acts as a danger-signaling receptor in glial cells and macrophages. Scientists have reported that by increasing the expression of P2X7R (Arulkumaran et al., 2011), hemin makes cells more sensitive to extracellular ATP (eATP). This ATP–P2X7R co-signaling axis is crucial for potassium ion efflux, which directly serves as the main initiating signal for assembling the NLRP3 inflammasome, thereby pre-activating the downstream inflammasome. Scientists have also reported that although the expression of P2X7R induced by hemin enhances the classical NLRP3 activation process (Dutra et al., 2014), hemin itself can also directly activate the NLRP3 inflammasome through mechanisms independent of P2X7R or eATP, due to the hemin-induced independence of the inflammasome. The cytoprotective enzyme hemin oxygenase−1 (HO−1) has been reported to provide negative feedback (Zhao et al., 2018). As a substrate, hemin induces HO−1 expression, whose activation mediates anti-inflammatory effects that can eventually dampen purinergic and inflammatory signaling cascades, thereby creating a negative feedback loop that limits further P2X7R-mediated cell death. In our study, we discovered that hemin induces microglial phenotypic transformation and upregulates P2X7R/NLRP3 expression in microglia, thereby regulating the P2X7R/NLRP3 pathway.

Following vessel rupture in ICH, the release of blood jets causes immediate tissue destruction (Al-Shahi Salman et al., 2018). Various endogenous molecules are released into the brain parenchyma, leading to cytotoxic and excitotoxic effects through neuroinflammation, exacerbation of oxidative stress, and disruption of cellular signaling pathways, ultimately resulting in secondary brain damage (Graeber et al., 2011). This secondary damage involves the formation of edema, inflammation, and cell death in the perihematoma region. There is mounting evidence that hemoglobin and iron released from hematomas contribute to both cell-autonomous and non-cell-autonomous neuronal damage (Keep et al., 2012). Moreover, microglial activation is closely associated with inflammation in secondary brain injury after ICH. Activated microglia play two distinct roles in ICH: anti-inflammatory microglia exhibit a protective role by phagocytosing cellular debris and harmful products, while pro-inflammatory microglia release pro-inflammatory cytokines (IL−1β, IL−6, TNF-α) that result in a strong inflammatory response (Lan et al., 2017). In the present study, we discovered that hemin induces microglial phenotypic transformation. Hemin upregulated the expression of P2X7R/NLRP3 in microglia, thereby regulating the P2X7R/NLRP3 pathway. Furthermore, the levels of the pro-inflammatory cytokines IL−1β, IL−6, and TNF-α increased noticeably following hemin induction. Additionally, the levels of the anti-inflammatory cytokines IL−10 and IL−4 increased significantly.

Research has revealed that vitamin D can influence various biological processes, including cell differentiation, the expression of neurotrophic factors, intracellular calcium signaling, neurotransmitter release, antioxidant activity, anti-inflammatory effects, stress responsiveness, and the regulation of genes/proteins crucial for the proper functioning of neurons (Eyles et al., 2013, McCann and Ames, 2008). Several studies have found a correlation between embryonic or neonatal vitamin D deficiency and an increased risk of developing neurodevelopmental disorders (McGrath et al., 2010, Eyles et al., 2018). Additionally, vitamin D deficiency in adults has been linked to certain neurodegenerative diseases (Kesby et al., 2011). Studies have also shown that vitamin D can enhance hematoma clearance and neurological recovery following ICH (Liu et al., 2022b). In the current study, we observed that vitamin D inhibits microglial phenotypic transformation toward the pro-inflammatory phenotype, promoting the anti-inflammatory phenotype and thus effectively suppressing microglial inflammation.

Scientists have reported (Fiebich et al., 2018) that Toll-like receptors (TLRs) initiate the innate immune response and prepare microglia for activation. The vitamin D signal acts on the TLR network (including TLR4 and TLR10) and its downstream signaling cascades. When the TLRs of microglia are activated by pathogens or damage-associated molecular patterns (DAMPs), vitamin D can inhibit the subsequent release of toxic reactive nitrogen and oxygen substances, thereby protecting neighboring neurons from inflammatory damage. Other scientists reported (Zhang et al., 2025, Lee et al., 2026a) that the NF-κB and mitogen-activated protein kinase (MAPK) pathways are classic drivers of neurotoxicity and pro-inflammatory microglial cell polarization. Through both genetic (vitamin D binding) and non-genetic mechanisms, calcitriol (active vitamin D) inhibits the phosphorylation of MAPK and blocks the nuclear translocation of NF-κB. This prevents the transcription of hundreds of inflammatory genes, keeping microglial cells in a baseline monitoring or tissue repair phenotype. Furthermore, scientists have reported (Zhang et al., 2023, Lee et al., 2026b, Cui et al., 2023) that phagocytosis and M2 polarization regulation differ from those of overactive M1-type microglia. M1-type microglia release toxic cytokines and cause the death of surrounding neurons, while M2-type microglia are specifically responsible for clearing waste and tissue repair. The vitamin D receptor (VDR) signaling pathway is necessary for inhibiting ischemia-induced neuroinflammation and promoting the transition to the M2 type. It upregulates specific surface markers (such as CD206) and regulates microglial phagocytosis, helping to clear protein aggregates such as β-amyloid and protecting the surrounding microenvironment from excessive inflammatory activation. Scientists also reported that, via the IL−10/suppressor of cytokine signaling 3 (SOCS3) axis (Boontanrart et al., 2016), vitamin D3 regulates microglial immune activation through an endogenous pathway. This pathway promotes the expression of the anti-inflammatory cytokine IL−10. When the level of IL−10 increases and binds to the IL−10 receptor of microglia, the expression of SOCS3 can then be upregulated. This forms a self-secreted anti-inflammatory feedback loop that can strongly inhibit the transcription of pro-inflammatory cytokines, including TNF-α, IL−6, and IL−12. Our research also found that in the presence of the P2X7R inhibitor A438079, vitamin D further enhanced the protective effect on microglial cells. This result may indicate that vitamin D has an additional anti-inflammatory effect independent of P2X7R, which is consistent with the aforementioned reports.

6P2X7R, a critical regulator of NLRP3 inflammatory signaling, has received significant attention for its role in mediating disease pathology (Di Virgilio, 2007). Mounting evidence demonstrates the involvement of P2X7R in various central nervous system diseases, such as ischemic stroke, neurotrauma, subarachnoid hemorrhage, neuropathic pain/epilepsy, and neurodegenerative diseases (Sperlágh and Illes, 2014, Bartlett et al., 2014). In an ICH model in rats, P2X7R blockers prevent activation of the NLRP3 inflammasome and limit brain damage (Feng et al., 2015). Furthermore, research has shown that endogenous hydrogen sulfide counteracts NLRP3 inflammasome-induced neuroinflammation by inhibiting P2X7R activity in rats with ICH (Zhao et al., 2017). In this study, we discovered that the levels of P2X7R and NLRP3 were significantly heightened in microglia induced by heme. However, the addition of vitamin D effectively downregulated their expression. Furthermore, the use of a P2X7R inhibitor significantly diminished the phenotypic transformation of microglia toward a pro-inflammatory phenotype and promoted their transformation toward an anti-inflammatory phenotype. Meanwhile, vitamin D treatment further amplified the effect of the P2X7R inhibitor on microglial phenotypic transformation. Overall, our findings suggest that vitamin D can inhibit the phenotypic transformation of microglia to a pro-inflammatory type by inhibiting the P2X7R/NLRP3 signaling pathway, thereby promoting their phenotypic transformation to an anti-inflammatory type. Meanwhile, vitamin D is readily available, safe, inexpensive, and well-tolerated. Our findings provide strong support for future clinical studies using vitamin D to treat ICH.

There are reports that, following ischemic stroke, damaged cells release large amounts of ATP, which acts as a danger signal to activate the P2X7R on microglial membranes. Activation of P2X7R induces a burst of ROS via NADPH oxidase and mitochondrial pathways (oxidative stress). Subsequently, ROS act as a key second messenger, promoting the dissociation and reassociation of thioredoxin-interacting protein (TXNIP) with the NLRP3 inflammasome, thereby driving its assembly and activation. The activated NLRP3 inflammasome further cleaves pro-caspase−1 into active caspase−1, thereby promoting the maturation and secretion of IL−1β and IL−18 and triggering an inflammatory cascade. Notably, IL−1β itself can stimulate neighboring cells to produce more ROS, while pyroptosis mediated by P2X7R releases additional ATP, forming a positive feedback loop (i.e., the ROS–P2X7R–NLRP3 axis) that exacerbates neuronal damage (Wu et al., 2012). Therefore, we believe that the ROS signaling pathway may be associated with the P2X7R–NLRP3 pathway. Therefore, targeting key nodes along this axis—such as ROS or P2X7R—may offer novel strategies to mitigate post-stroke inflammatory injury. Given vitamin D’s antioxidant properties (Vázquez-Lorente et al., 2024), this could also represent a promising direction for our next experimental investigation.

## 5 Conclusion

Overall, our findings suggest that vitamin D can regulate microglial phenotypic transformation through the P2X7R/NLRP3 signaling pathway, thereby promoting anti-inflammatory and inhibiting pro-inflammatory phenotypic transformation. Our findings provide strong support for future clinical studies using vitamin D to treat ICH.

## CRediT authorship contribution statement

**Bensi Zhang:** Writing – review & editing, Investigation, Funding acquisition, Data curation. **Xiujun Zhang:** Writing – review & editing, Writing – original draft, Visualization, Validation, Methodology, Investigation, Formal analysis, Conceptualization. **Manussabhorn Phatsara:** Writing – review & editing, Supervision, Funding acquisition, Conceptualization. **Suteera Narakornsak:** Writing – review & editing. **Natnicha Thammarangsee:** Writing – review & editing. **Chun Shi:** Writing – review & editing. **Rungusa Pantan:** Writing – review & editing. **Waleephan Treebupachatsakul:** Writing – review & editing.

## Funding

This study was supported by the 10.13039/501100010731Faculty of Medicine, Chiang Mai University (ANA−2568–0081), the 10.13039/501100008871Department of Science and Technology of Yunnan Province (NO.202101AN070028), and the Li Yunqing expert workstation of Yunnan Province (No.202005AF150014).

## Declaration of Competing Interest

The authors declare that they have no known competing financial interests or personal relationships that could have appeared to influence the work reported in this paper.
